# *In Silico* Profiling of Clinical Phenotypes for Human Targets Using Adverse Event Data

**DOI:** 10.3390/ht7040037

**Published:** 2018-11-23

**Authors:** Theodoros G. Soldatos, Guillaume Taglang, David B. Jackson

**Affiliations:** Molecular Health GmbH, Kurfuersten Anlage 21, 69115 Heidelberg, Germany; soldatos@molecularhealth.com (T.G.S.); guillaume@taglang.org (G.T.)

**Keywords:** computational biology, large-scale approaches, systems pharmacology, adverse events, side-effects, outcome analytics, real world data, mode of action, clinical phenotypes, phenotypic screening, drug safety prediction

## Abstract

We present a novel approach for the molecular transformation and analysis of patient clinical phenotypes. Building on the fact that drugs perturb the function of targets/genes, we integrated data from 8.2 million clinical reports detailing drug-induced side effects with the molecular world of drug-target information. Using this dataset, we extracted 1.8 million associations of clinical phenotypes to 770 human drug-targets. This collection is perhaps the largest phenotypic profiling reference of human targets to-date, and unique in that it enables rapid development of testable molecular hypotheses directly from human-specific information. We also present validation results demonstrating analytical utilities of the approach, including drug safety prediction, and the design of novel combination therapies. Challenging the long-standing notion that molecular perturbation studies cannot be performed in humans, our data allows researchers to capitalize on the vast tomes of clinical information available throughout the healthcare system.

## 1. Introduction

Deciphering molecular mechanisms connecting disease phenotypes to the underlying genotypes remains one of the most important endeavors in science and medicine. Hidden within this molecular conundrum lies the knowledge that can power rational approaches to the detection, prevention and treatment of human disease. However, despite the enormous implications for our health and well-being, we still rely heavily on evidence from genetic and pharmacological perturbation experiments in model systems, such as cell-lines and model organisms. While certainly contributing to the identification of the key molecular protagonists of human disease, model systems suffer important limitations, the most fundamental of which remains their general lack of absolute congruence with the human condition that they are used to characterize [1]. While this is understandable given the enormous complexity and cell-type specificity of biological systems, it does suggest that new levels of therapeutic innovation might be achieved through development of methods that allow us to decipher molecular mechanisms directly from human specific data.

This is elegantly demonstrated by results from the TCGA project, where molecular data and phenotypic information have been analyzed to decipher novel targets and prognostic classifiers. Analysis of the TCGA endometrial carcinoma dataset, for example, has brought important new insights into the molecular nature of this disease [2], including the discovery of a new classification system based on four prognostically significant subgroups. Indeed, it can be argued that most recent clinical advancements emerge from analysis of patient-derived molecular data. However, vast tomes of clinical information available throughout the healthcare system may remain underutilized, unless it becomes possible to derive molecular insights and hypotheses from these data.

**Figure 1:**
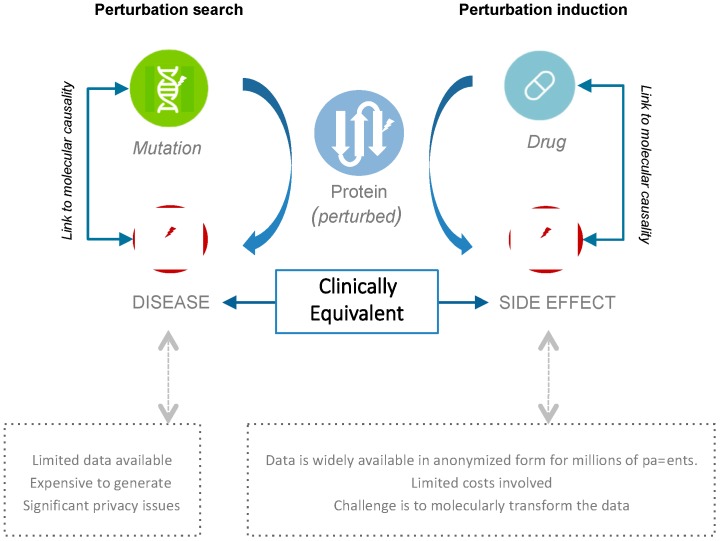
Perturbation studies are key to understanding human disease and side effects. Recent advances in high-throughput molecular technologies have revolutionized our ability to characterize the molecular foundations of human phenotypes. However, costs and privacy concerns remain a key impediment to the broad-scale analysis of such data. Our approach allows deciphering molecular mechanisms directly from accessible real-world clinical data: The phenotypic read-outs from drug interventions typically involve desired (e.g., cure) and/or undesired phenotypic outcomes (e.g., side-effects).

One key challenge with using such sources of accessible human phenotype data, like electronic clinical reports, is the dearth of associated molecular data. We asked whether it might still be possible to connect a patient’s clinical phenotype to molecular causation using the fundamental fact that physicians have been performing perturbation-studies on their patients for decades (Figure 1). From a molecular perspective, drugs are prescribed to modulate the function of disease-related molecular entities (typically proteins), with the goal of counteracting disease-causing mechanisms. Thus, it stands to reason that if we were able to link the molecular functions targeted by therapeutics to resultant side-effect phenotypes, and this for millions of patients, we should be able to computationally dissect the molecular mechanisms underlying these effects. This would enable production of datasets that might be of value to the molecular characterization of human disease directly from real world phenotype data.

**Figure 2:**
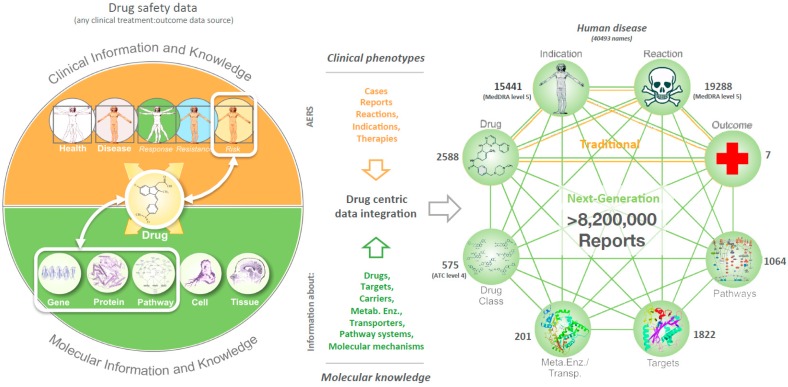
To enable the molecular analysis of drug induced clinical effects our approach adopts the simple premise that drugs induce side effects through perturbation of protein function. Drug-target knowledge can then be used to dissect the molecular basis of human disease. To achieve this, we built a drug-centric data integration process that currently combines observational clinical data for 8.2 million drug-safety reports with molecular information about drugs and their targets. The applied drug-centric data integration approach expands on traditional pharmacovigilance approaches to provide a new model for safety prediction and assessment through mechanism- and molecular-based analysis of drug induced phenotypes. Our target-reaction collection is a high dimensionality resource that to our knowledge contains more target-phenotype associations than any other relevant collection to-date. In specific, a multitude of 1.8 million target-reaction associations were characterized and can be explored via our dataset (Supplementary files 1, 2, 3, 4).

With this goal in mind, we developed a methodology for the molecular characterization of datasets providing drug-perturbation and phenotype observations. We analyzed data from the FDA Adverse Event Reporting System (FAERS) that contains millions of electronic adverse event (AE) case-reports, which document individual patients in de-identified format, their disease, and the side-effect consequences of co-reported drug treatments. While such data provides a direct connection between drugs and their phenotypic consequences for millions of patients, public resources such as the DrugBank [3] database are also available, which describe the targets of marketed and developmental drug therapies. It was therefore possible to connect phenotypes directly to targeted proteins/genes using a drug-centric data-integration process that maps drugs in each FAERS case-report, to their associated targets (Figure 2). Targets can further be contextualized by their pathway membership, permitting broader molecular connectivity with associated phenotypic outcomes. Using this approach, we extracted 1.8 million associations of clinical phenotypes to 770 human drug-targets.

In this work we describe our approach, the content of the produced phenotypic profiling of human targets, we present validation results, and give examples that demonstrate some of the key analytical utilities. In specific, we discuss analytical modality concepts enabled by this approach, such as the exploration of clinical phenotypes caused by perturbation of human targets, the prediction of side-effects of new drugs, and the ability to perform virtual perturbation experiments by systematically comparing drug or target profiles. Overall, we find that insights derived directly from patient/clinical data can be more specific, compared to other methods.

## 2. Materials and Methods

### 2.1. Connecting Clinical Phenotypes to Molecular Knowledge

Drugs typically modulate protein function to produce both desired (e.g., curative) and undesired (e.g., side-effects) clinical phenotypes. Given that side effects are essentially drug-induced diseases, we proposed that it would be possible to dissect the molecular basis of both side effects and diseases by analyzing phenotype data using drug-target knowledge. We achieved this via a drug-centric data integration process that combined clinical data for millions of patients with underlying molecular knowledge (targets, metabolizing enzymes, etc.). Results of this methodology are available as Supplementary files.

### 2.2. Data Integration

We developed a drug-centric data integration process that combines clinical data for about 6.8 million patients from FAERS (2000–2016), with the molecular knowledge in the DrugBank [3] database. This FAERS dataset holds about 8.2 million drug safety reports containing information about each patient’s treatments, and disease indications and side-effects (termed reactions) coded using the MedDRA ontology [4]. The DrugBank database contains information about the molecular targets of marketed and developmental drugs. Matching treatments in each safety report to associated knowledge in DrugBank is complicated by text mining considerations [5], such as the non-standardized drug names in FAERS. Key challenges lie in the fact that FAERS medication references may refer to one or to many drugs, may contain spelling errors or abbreviations, or may mention lump categories (e.g., vitamins, or antibiotics). We therefore developed a specialized mapping process that uses both drug synonym dictionaries and computational linguistic strategies to address these issues.

In terms of process, we employ a stepwise procedure. First, we link all FAERS names that are identical with a drug synonym. Then, for those medications that have not been mapped, we identify parts that are identical with or similar to a drug synonym, by using regular expressions. The process stops when the examined phrases differ by more than 5 characters. These features allow to compensate for common spelling variants or errors, but also to reduce the search space. In each phase of the mapping procedure a blacklist is used to exclude irrelevant terms (e.g., UNKNOWN, UNK) from being matched. By applying this mapping procedure, we successfully linked about 90% of all drug names mentioned in FAERS reports to single unambiguous therapeutic species, leading to at least one mapping for about 95% of all FAERS reports.

Further levels of molecular integration can be achieved by mapping all DrugBank proteins to their respective pathways within the Reactome [6,7] and PID (NCI-Nature & BioCarta) [8] resources. We have also integrated the ATC ontology from the WHO [9] to allow exploration and comparison of hierarchical drug classes. As a result, 8.2 million patient reports detailing drug-induced phenotypes could be linked to information about nine entity types: 15.4 K indications, 19.3 K reactions, seven clinical outcomes, and 2.6 K drugs, to 1.8 K targets, 201 metabolizing enzymes, 103 transporter proteins, 1 K pathways, and 881 ATC drug classes. Here we present results regarding targets, and reactions linked to at least 500 AE cases each (see Supplementary files 1, 2, 3, and 4).

### 2.3. Characterization of Relationships

Next, we sought to characterize the relative degree of statistical association between dugs and targets with reactions. For this purpose, we employed the proportional reporting ratio (PRR), an established measure of disproportionality in pharmacovigilance (Table 1). PRR gives an indication for the relative congruence of pairwise entity relations as based on their co-occurrence in subsets of AE data [10]. 

Characterization of relationships in this way helps not only to identify known or novel relationships but also to perform relative comparisons. Furthermore, as the characterization of relationships spans all entities, a bidirectional study is possible: Physiological effects can be examined with respect to underlying mechanisms, or conversely, one can examine potential clinical results when specific molecular systems are perturbed. Moreover, previously reported events can be used to indicate (or predict) effects of potential therapies helping avoid erroneous prescriptions and toxic combinations.

### 2.4. The Collection of Relationships

We focused on 770 targets that were linked to at least 500 AE cases and were not listed in DrugBank [3] as also metabolizing enzymes, carriers, or transporters of other drugs. Phenotype co-occurrences of these ‘clean’ targets exceeded 12.9 million pairwise associations (i.e., target co-mentioning in AEs with each of seven clinical outcomes or indications and reactions expressed at any level of the MedDRA hierarchy). Inspecting phenotypes at higher levels of the MedDRA hierarchy helps compensate for occasional redundancy attributed to the reporting of reactions in AEs by using similar terms or the reporting of reactions that are highly related (e.g., ‘Coronary artery disease’, ‘Coronary artery thrombosis’, and ‘Coronary artery restenosis’). Association of targets to reaction classes of MedDRA level 2 alone gave rise to more than 230,000 relationships (Supplementary file 1). While PRR has become a standard metric for the evaluation of safety signals, we also considered relevant statistical significance be reflected by Fisher’s exact test *p*-values and respective *q*-values [11]. For reference, we provide in Supplementary files 2, 3, and 4 all 1,601,362 co-occurrences of targets with reactions expressed at MedDRA level 4. Extracted targets and reactions were limited to those mentioned in at least 500 AEs each.

### 2.5. Presented Validation Examples

We also present validation results demonstrating analytical utilities of the approach, including drug safety prediction and the design of novel combination therapies.

#### 2.5.1. Prospective Prediction of Side Effects

To validate the hypothesis, we used a dataset of older AEs that contained 2.4 million patients (AE cases from the 2000–2011 subset of FAERS). We examined adverse reactions and summarized them in terms of MedDRA level 1 categories. AE relationships were characterized using as measure the PRR metric.

#### 2.5.2. Hypothesis Testing for Improved Use of Drugs

*Beta-adrenergic receptor* (*BAR*) activation affects processes involved in the progression of various cancer types [12,13]. We asked whether *BAR* antagonism could reduce skin cancer related mortality and therefore compared the clinical outcomes of two *BAR* activity modulations in a skin cancer patient population. The skin cancer cohort of this study comprised of those patients whose indication linked to the MedDRA (level 2) term ‘Skin neoplasms malignant and unspecified’. Beta-blocking agents were defined with the help of the ATC classes C07AA, C07AB, and C07AG. Death occurrence was based on the reporting of the respective outcome. In absence of *BAR* antagonism, reported deaths occurred in 4059 out of 17,177 cases (23.6%), whereas with *BAR* inhibition deaths were reported in 241 out of 1308 cases (18.4%). The result for reduced skin cancer mortality with co-medication of beta-blockers is significant with Fisher’s exact *p*-value = 0.000012.

### 2.6. Benchmarking

We relied on a benchmark set used previously in [14] and manually mapped 53 known protein–effect pairs [15] to our data. For the extraction we used the following criteria:

(a) To ensure specificity we primarily considered reactions described at MedDRA level 4. In many cases AEs mentioned exactly a certain term as reported in the original dataset (e.g., ‘Tachycardia’), whereas other times an effect could only be captured in AEs via higher MedDRA level descriptions (e.g., ‘salivation’ and ‘Salivary gland disorders NEC’). In some other cases this strategy led to the identification of reaction terms too specific and with scant occurrence in AEs (e.g., ‘Hypophosphatemia’ or ‘Hypocalcaemia’).
(b) We searched only for reactions: The physiological effects expected to occur when compounds hit the listed targets of [15] may be characterized with increased PRRs in AEs, however sometimes they may only reflect a condition and not an adverse reaction.
(c) We searched for up to two synonyms per case: Sometimes the same effect may have been declared via its synonyms or in similar spelling (e.g., ‘anemia’ and ‘anaemia’). In other cases, alternative names were searched to describe generic phrases of the original dataset (e.g., both ‘hypertension’ and ‘hypotension’ were searched for the originally termed effect ‘blood pressure changes’).
(d) We also did not consider effects of proteins that were not targets or had no drugs reported in AEs.

The results of the manual mapping process can be found in Supplementary file 3, including cases that could not be exactly mapped (e.g., ‘cell proliferation’), cases better described in higher levels (like ‘Salivary gland disorders NEC’ instead of ‘salivation’), or cases when more than one terms may describe a condition. In those cases, phenotypes described in higher MedDRA levels would likely have been more appropriate to capture a certain valid association. This highlights the importance of accessing and assessing phenotype relationships at different levels/classes. Benchmarking challenges that could be attributed to the nature of FAERS reporting (i.e., the way terms are reported and coded) included redundancy and that a reaction might sometimes reflect an indication.

Overall, our search was manual and not exhaustive. Therefore, some of the selected terms may not be the most appropriate to highlight a certain target’s relation to a phenotype as other synonyms may exist that have a better (higher/lower) signal. For the comparison task described next, we focused only on fifty derived heart and blood effects. We also kept three effects that were specific and with stronger signals, co-reported in more than 2500 AEs with respective targets (namely, ‘Dry mouth’, ‘Electrocardiogram QT prolonged’, and ‘Dyskinesia’).

### 2.7. Comparison of Target-Reaction Signals

We contrasted molecular analysis of phenotypes from AE patient prescriptions with methods that annotate proteins with side-effect information derived from phenotype associations of the drugs that target them. To represent the latter methods, we used the approach of [14] and for each reaction–target pair we counted the number of drugs that were co-reported with each effect, that target the protein, that do both, and neither. Then, we calculated respective PRRs and the *p*-value for overrepresentation using Fisher’s exact test. The results indicate that some of the benchmarked relationships would have been dismissed by this approach (Supplementary Figure S1; Supplementary file 5). PRR-signals calculated directly from targets’ AEs were generally more definitive which could be attributed to the fact that each drug individually may be mentioned in some AE observations but not (necessarily) in all cases that a certain target may have been perturbed. Our approach to characterize the molecular landscape of patient prescriptions allows thus extracting relationships more directly. To assess the variability of associations derived indirectly via drugs we listed in Supplementary file 5 also the respective maximum, average, and minimum drug-reaction PRR values, among the drugs targeting each target-reaction pair. 

### 2.8. Data Availability

Phenotype annotations between targets and reactions are provided at Supplementary files 1, 2, 3, and 4.

## 3. Results

Our molecular characterization approach enables analysis and comparison of the phenotypic consequences associated with the perturbation of human proteins and/or to identify potential molecular mechanisms associated with distinct (or related) clinical phenotypes. It also enables the definition of patient-specific disease models in a manner that permits the dissection of underlying molecular mechanisms. In terms of numbers, FAERS (2000–2016) captured 15,441 indications (i.e., clinical phenotypes prior to drug treatment) and 19,288 AE reactions (i.e., clinical phenotypes after drug treatments).

### 3.1. Phenotypic Profiling of Human Target Proteins

Using drug-to-target data from DrugBank [3] and the treatment information provided for each patient in 8.2 million FAERS reports, 1822 human proteins were mapped to associated clinical phenotypes. Of these, 770 targets linked to at least 500 AE-cases and were listed in DrugBank [3] only as targets of drugs (and not as also metabolizing-enzymes, carriers, or transporters of other drugs). Quantifying the scope and diversity of phenotype observations (including indication and reaction names expressed at any level of the MedDRA hierarchy, and outcomes), these 770 targets gave rise to more than 12.9 million target-phenotype associations (Supplementary Table S1). Described at MedDRA level-2 terms only, 333 reactions were mentioned in 230,841 combinations with these targets (Supplementary file 1). Of these associations, 155,820 (67.5%) were statistically significant (*q* < 0.05), while 42,284 (18.3%) connected via at least ten AE-cases and also have proportional reporting ratio (PRR) value ≥ 2. Another 1,601,362 associations (Supplementary files 2, 3, and 4) between the 770 targets and 2803 reactions (MedDRA level-4 names) were characterized: 809,451 (50.5%) were found statistically significant (*q* < 0.05) and 290,440 (18.14%) appeared in at least ten AEs and have PRR ≥ 2. These results highlight the specificity of many of the detected target-phenotype associations. They also emphasize the importance of exploring and assessing candidate associations at different levels of phenotype and target classes (Supplementary Figure S1).

Another aspect of this computational approach is that it enables systematic analysis of potential molecular players involved in human disease. The community can use our data to identify potential molecular protagonists across clinical phenotypes via a simple comparison strategy, permitting thus the step-by-step generation and dissection of molecular hypotheses.

### 3.2. Using the Approach: Examples and Perspectives of Analytical Strategies

Our approach allows linking drug-induced phenotypes to potential underlying molecular etiologies. Phenotypes can thus be analyzed and compared at different levels of phenotype and target classes (example at Supplementary Figure S1), at the level of any molecular perturbation (example at Supplementary Table S2), or specific clinical and molecular feature (examples at Supplementary Figures S2 and S3).

Importantly, comparative analytics permit the step-by-step generation and dissection of molecular hypotheses for assessing the functional association of targets with clinical phenotypes. For example, the most strongly associated reaction with *EGFR* perturbation was ‘dermatitis acneiform’ (extracted from Supplementary files 2, 3, and 4). This observation is consistent with current knowledge about the function of *EGFR* in epithelial biology [16,17,18] and demonstrates how even such a simple comparison strategy can help decipher phenotypic consequences of human target perturbation by using our resource.

Building on this principle we also demonstrated that it is possible to explore phenotypic consequences that result from different functional/activation states of a target (Supplementary Figure S1): We focused on a selection of targets whose function can be modulated in this way (activation versus antagonism) and determined reaction profiles with respect to the mechanism (agonism/inhibition) of the related drugs, using PRRs. Comparison of the phenotypic profiles reveals in each case differences between classes of targets and their functional modulation.

It is this, perhaps, one of the most important properties enabled by our approach for profiling individual patient prescriptions: The ability to define and investigate cohorts with specific characteristics (such as conditions, therapies, molecular entities) and compare them against other groups of patients. This allows performing systematically virtual perturbation experiments and identifying features or patterns by using a variety of methods, such as refined statistics, systems biology, and machine learning techniques. These perspectives do not directly compare to true perturbation studies (e.g., clinical trials, examination of side effects, experimental controls, etc.), but rather complement them. Real world application of our methodology has been shown in several instances to effectively help generate or validate hypotheses in terms of retrospective analysis of molecular or therapeutic properties e.g., [19,20].

### 3.3. Validation Results

To demonstrate some of the several predictive capabilities enabled by our approach we present two examples:

1. Prospective prediction of drug side effects. We asked whether it might be possible to predict AE-profiles of novel drugs. Examining the reaction profiles of tyrosine kinase inhibitors (TKIs) revealed that Sorafenib is more strongly associated with dermatological reactions than Sunitinib (Supplementary Table S2). Indeed, skin-related AEs are a common form of side-effects observed in patients treated with TKIs [16,17,21,22]. However, while these two TKIs share a common target-inhibition profile [23,24,25,26], Sorafenib additionally inhibits *BRAF*. We capitalized then on the Sorafenib-specific side-effect difference to hone in on the *BRAF*-specific clinical effects and hypothesized that the reaction profiles of other *BRAF* inhibitors may also include skin-related AEs. To test whether we could have predicted this relationship for the *BRAF*-specific inhibitor Vemurafenib, we used a data slice of all patient-cases reported prior to Vemurafenib’s FDA-approval in August 2011. Through this prospective-retrospective analysis we found that *BRAF* perturbation was strongly related to dermatologic reactions (Supplementary Figure S2)—thus predicting the primary side-effects observed in Vemurafenib’s phase-3 trial [27] (NCT01006980). Indeed, predicted skin-related AEs are consistent with BRAF pathway findings from recent studies and other independent work [28,29,30,31]. Importantly, this clinical effect is also included in Vemurafenib’s FDA-label [32].
2. Rational prediction of combination therapies. We also assessed whether we could identify combinations of targets that may improve therapeutic outcomes in patients. We reasoned that AE outcomes and in particular “death rate”, might be used as surrogate marker for treatment efficacy. Using prior knowledge about the association between biobehavioral stress and tumorigenesis [12,13], we investigated whether perturbation of *beta-adrenergic receptor* (BAR) function in cancer patients might result in lower patient mortality. We examined the phenotypic consequences of *BAR* activity modulation by comparing two similar cohorts of skin cancer patients—one arm with and one without inhibition of *BAR* activity (Supplementary Figure S3). In absence of *BAR* antagonism, reported deaths occurred in 23.6% cases, whereas with *BAR* inhibition deaths were reported in 18.4% cases. These results suggested that co-medication of *BAR* blockers may be associated with reduced mortality of skin cancer patients, and were supported by subsequent in vitro and in vivo studies which demonstrated the role of *BAR* in tumor growth and stress-response signaling via *SRC* activation in cancer cells. Further analysis revealed that mortality was reduced across major cancer types in patients where *BAR* signaling is inhibited with beta-blockers and identified *BAR* inhibition as a potential combinatorial route to anti-cancer treatment [33]. In support of these observations, a recent phase-2 pilot-study (NCT01265576) examining clinical effects of beta-blocker usage in hepatocellular carcinoma (HCC) patients with VT-122 (combination of the non-selective beta-blocker Propranolol and the COX2-selective Etodolac) demonstrated potential anticancer effects when co-administered with Sorafenib by increasing therapy duration and overall survival, as compared to HCC patients treated with Sorafenib alone [34]. Our example study emphasizes the importance of clinical phenotype profiling at the level of target perturbations, but also provides a potentially novel approach to drug repositioning, via rational design of combination therapies.

### 3.4. Benchmarcking and Comparison

We also benchmarked our findings against an independent dataset of known target–reaction effects, derived manually from [15] (Supplementary file 5). Overall, we find that characterization of target-reaction relationships are more conclusive when relying directly on patient/clinical data, as compared to methods that indirectly combine separate sources of drug side-effect data with molecular information e.g., [14,35]. Specifically, 56% more target-reaction relationships were recapitulated from the benchmark-set when we contrasted PRR-signals calculated over targets’ AE-cases directly against those derived via respective drug-reaction associations. PRR-signals calculated directly from target AEs were generally more definitive, an effect likely attributable to the broader scope of these observations, as compared to those for each drug separately. In return, this allows identifying target-specific phenotype effects that may otherwise remain masked, undermined by side-effect consequences of the combined co-perturbation of one drug’s targets.

These results highlight the importance of direct characterization of target-phenotype relationships from large amounts of patient clinical data. However, an absolute comparison to similar methods that rely on other data is neither fair nor straightforward, and we believe that our results and results derived from different methods should be used in complementary ways. Also, our experience calls for the definition of a more proper benchmark to systematically compare similar approaches.

## 4. Discussion

Our dataset is the first and—to the best of our knowledge—currently only that permits molecular analysis of real-world phenotypes using accessible clinical data. It is also unique in that it enables scientists to quickly develop testable molecular hypotheses directly from human-specific observations, as opposed to model-organism data.

Previous systems-biology efforts to combine molecular information with phenotypes e.g., [14,36,37] relied primarily on side-effect information coming from labels e.g., [38,39]. AEs comprise an augmented source capturing real-world scenarios regarding drug use and combinations, phenotypes and conditions not studied in clinical-trials, and also include information for many more patients. However, FAERS data alone comes with limitations such as the lack of standardization [40]. Our work is an advance over previous target-phenotype association studies in that it addresses these shortcomings and provides a standardized approach to linking drug-induced AEs to potential underlying molecular etiologies. Specifically, with this work we combined previous efforts to, in addition, link phenotypes to the underlying molecular landscape, based on the annotation of AE patient-cases themselves. This characteristic of our approach permits phenotypes to be analyzed and compared at the level of any molecular perturbation or specific clinical and molecular feature (including drugs, drug-classes, targets, metabolizing enzymes, etc.). Furthermore, our strategy to characterize target-phenotype associations directly from their occurrence in clinical data, as opposed to using indirect drug-centric properties or observations e.g., [38,40], allows enriching existing integrative systems approaches with protein-perturbation datasets of additional levels of evidence [41,42].

Despite current limitations in known drug-target knowledge, we presented a number of case studies validating key utilities of our approach. We demonstrated how it could be used to identify potentially novel molecular players involved in diseases and illustrated how such molecular profiling can aid drug-safety prediction. Moreover, we demonstrated that it could be used to aid the identification of novel drug or target combinations associated with the improvement of specific phenotypes or outcomes. Importantly, comparison analytics over these results permit the step-by-step dissection of hypothesis for molecular causation and influence of individual targets on clinical phenotypes. In specific, our collection of target-phenotype associations can help identify targets associated with the exacerbation of specific phenotypes and/or an increase in their overall prevalence.

Integrating AE-data with molecular knowledge also expedites the identification of safety problems via a prospective approach. It provides drug-safety scientists with the unique ability to not only identify safety signals, but also assess whether any biomolecular rationale might exist to support traditional pharmacovigilance-based statistics. This is critical to both regulators and pharmaceutical companies, providing a more transparent assessment of whether a drug might be functionally involved in the emergence of a drug-safety issue. In this regard, our results continue to be utilized by clinical pharmacologists at the FDA in the process of drug relabeling due to newly identified safety issues [43,44].

In terms of future developments, we envisage a number of important data and analytical enhancements. We aim to combine AEs together with additional observational data and real-world evidence. We also expect that leveraging chemistry and genotype information will help towards the development of broader systems approaches. We hope that our work will provide additional context and scope to the efforts to interpret and model human disease and invite our dataset to serve as foundation for an array of experiments, including analyses complementing data generated through direct characterization of patient genotypes.

## Figures and Tables

**Table 1:** For a drug (D) or a target (T) and an event (E) the PRR metric is defined as the value of *a*(*c* + *d*)/*c*(*a* + *b*), based on the following contingency matrix.

AE Cases	Event (E)	Not E	Totals
**D (T)**	*a*	*b*	*A* + *b*
**Not D (T)**	*c*	*d*	*C* + *d*
**Totals**	*a* + *c*	*b* + *d*	*N* = *a* + *b* + *c* + *d*

## Supplementary Materials

The following are available online at http://www.mdpi.com/2571-5135/7/4/37/s1. Supplementary file 1: Association of targets to reaction classes of MedDRA level 2; Supplementary files 2, 3, and 4: Co-occurrences of targets with reactions expressed at MedDRA level 4; Supplementary file 5: Benchmark details and results; Supplementary file 6: Support tables and figures.
