# Supplementary material for: In Silico Profiling of Clinical Phenotypes for Human Targets Using Adverse Event Data
Source: High Throughput. 2018 Nov 23;7(4):37. doi: 10.3390/ht7040037 (PMC6306940; doi:10.3390/ht7040037)
Supplement: Supplementary file 1 [file high-throughput-07-00037-s001.zip › high-throughput-385126-suppl-final/Supplementary_File_6_Figures_and_Tables.docx]

Supplementary Materials: *In Silico* Profiling of Clinical Phenotypes for Human Targets Using Adverse Event Data

Theodoros G. Soldatos, Guillaume Taglang and David B. Jackson

**Table S1. Number of associations observed in AEs.** The second column lists the number of phenotypes co-reported in AEs with the selected 770 ‘clean’ targets, while the third column lists the respective number of pairwise target-phenotype associations reported in the dataset. Indications and reactions are reported in FAERS using MedDRA terms (lowest level 5; see italic font). Using the MedDRA hierarchy, target-phenotype associations can be described at different MedDRA levels (ML) consolidating thus redundant synonymous observations, but also allowing for the exploration of associations over variable resolution of phenotype classes.

| **Phenotype** | **Names** | **Associations** |
| --- | --- | --- |
| **Outcomes** | 7 | 5293 |
| **Reactions (ML 1)** | 26 | 20005 |
| **Reactions (ML 2)** | 333 | 230841 |
| **Reactions (ML 3)** | 1682 | 894631 |
| **Reactions (ML 4)** | 15561 | 3685624 |
| ***Reactions* (ML 5)** | *18801* | 4069826 |
| **Indications (ML 1)** | 26 | 19735 |
| **Indications (ML 2)** | 333 | 192500 |
| **Indications (ML 3)** | 1633 | 600597 |
| **Indications (ML 4)** | 10812 | 1508343 |
| ***Indications* (ML 5)** | *14833* | 1717270 |
| **Total** | | **12944665** |

**Table S2. Association of reaction rash to TKIs and their targets within the context of NSCLC and renal neoplasms**. Rash is a common side effect of TKI treatment in some cancer types (including NSCLC and renal neoplasms) and considered as marker of TKI treatment success. Here, we report on four TKIs approved for those indications that have similar target-profiles with each other: namely, Erlotinib and Gefitinib (within the context of NSCLC), Sorafenib and Sunitinib (within the Renal neoplasms context). We observe that rash as reaction relates strongly to Erlotinib and Gefitinib both within the NSCLC context (PRR*) and across all the adverse-event set (PRR). Rash however relates more with Sorafenib than Sunitinib (within both the renal neoplasms context and across all adverse-events). We also looked at the profile of reaction rash with respect to the main therapeutic targets of each TKI, within the context of each indication (PRR*) and across all the adverse-event set (PRR). We find that *EGFR*- and *BRAF*-perturbations relate strongly to this dermatological reaction. We then focused on the difference between the rash-profiles of Sorafenib and Sunitinib. This pattern may be attributed to differences in their molecular profile, leading to the hypothesis that *RAF*-specific inhibitors may also include skin-problems among their side effects. Later, we assessed whether we could have predicted rash as side effect of the *BRAF*-specific inhibitor Vemurafenib based on a data set of adverse-events older than Vemurafenib’s FDA approval (see next, Supplementary Figure S2).

| **Drug** | **PRR*** | **P-value** | **Cohort (Indication)** | **PRR** | **P-value** | **Cohort (Indication)** | **ATC** |
| --- | --- | --- | --- | --- | --- | --- | --- |
| Gefitinib | 1.59 | <0.00001 | Non small cell lung cancer | 4.1 | <0.00001 | Any | L01XE02 |
| Erlotinib | 4.37 |  |  | 5.32 |  |  | L01XE03 |
| Sunitinib | 0.66 | <0.00001 | Renal neoplasms | 1.32 |  |  | L01XE04 |
| Sorafenib | 3.97 |  |  | 4.15 |  |  | L01XE05 |
| **Target** | **PRR*** | **P-value** | **Cohort (Indication)** | **PRR** | **P-value** | **Cohort (Indication)** | **TKI drugs** |
| EGFR (P00533) | 6.13 | <0.00001 | Non small cell lung cancer | 4.05 | <0.00001 | Any | Erlotinib, Gefitinib |
| VEGFR1 (P17948; also known as FLT1) | 1.03 | >0.05 (not) | Renal neoplasms | 1.97 |  |  | Sunitinib, Sorafenib (minor) |
| VEGFR2 (P35968; also known as KDR) | 1 |  |  | 2.03 |  |  | Sunitinib, Sorafenib |
| VEGFR3 (P35916; also known as FLT4) | 1.07 |  |  | 1.97 |  |  | Sunitinib, Sorafenib |
| KIT (P10721; also known as cKIT) | 1.26 | <0.05 |  | 2.13 |  |  | Sunitinib, Sorafenib |
| PDGFRB (P09619) | 1.27 |  |  | 1.95 |  |  | Sunitinib, Sorafenib |
| PDGFRA (P16234) | 0.52 | <0.00001 |  | 1.49 |  |  | Sunitinib |
| FLT3 (P36888) | 1.59 |  |  | 2.41 |  |  | Sunitinib, Sorafenib |
| RET (P07949) | 3.81 |  |  | 3.8 |  |  | Sunitinib, Sorafenib (minor) |
| BRAF (P15056) | 3.97 |  |  | 4.02 |  |  | Sorafenib |
| RAF1 (P04049; also known as cRAF or Raf-1) | 3.96 |  |  | 3.73 |  |  | Sorafenib |
| CSF1R (P07333) | 0.66 |  |  | 1.43 |  |  | Sunitinib |

**Figure S1. Strategies associated with the large-scale phenotypic profiling of human-targets from patient clinical data.** (a) Comparative entity-analysis permits exploration of clinical phenotypes caused by perturbation of human-targets: e.g., log_10_(PRR) comparison of tyrosine kinase associations with different reaction-categories reveals involvement in diverse functions. Using the resolution provided by different target- and reaction-classes helps investigate signals further at variable levels of abstraction – Supplementary files 1, 2, 3, and 4 allow the systematic screening of such target-specific phenotype associations. (b) Comparison of approaches to characterize the molecular landscape of phenotypes. We compared signals for known target-reaction associations (Supplementary file 5) extracted directly via AEs (upper) with signals calculated indirectly, by using drug-reaction co-occurrences (middle). Side-by-side comparison of hypertension and hypotension results (bottom) reveals that characterization derived from AEs directly is more definitive (higher PRRs, smaller confidence intervals (95% CI)). Notably, some of those associations might have been excluded from further analysis (white squares indicate associations found not significant by Fisher’s exact test; *p*-value > 0.05). (c) Perturbation studies are key to understanding human disease and side-effects. Model-based approaches (upper) to identifying the molecular underpinnings of human disease are similar to the prevalent process of perturbation-search where diseased patients and healthy individuals are compared for molecular-level differences. Our approach enables a new strategy based on the induction of target-specific perturbation using drug-treatments (middle). Comparison of targets and their agonism/antagonism modulation-states (lower) reveals in many cases dissimilar phenotypic consequences.


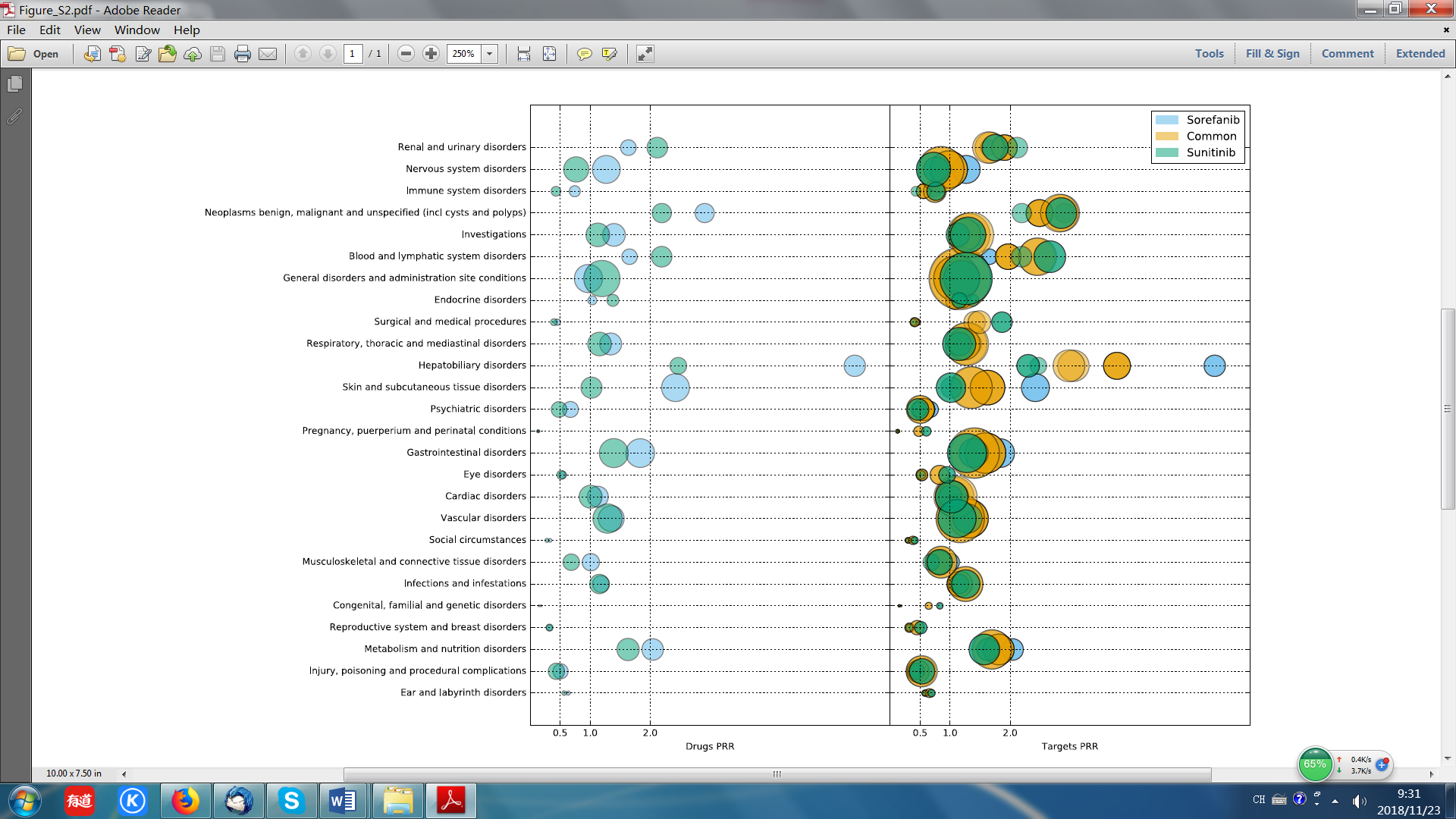


**Figure S2. Comparative drug-safety analysis for prospective prediction of side effects.** We assessed whether we could have predicted that dermatological reactions should be included in the side-effect profile of Vemurafenib. First, we characterized relationships (x-axis) between adverse-event (AE) reactions (y-axis) and Sorafenib, Sunitinib, and their targets, over a dataset of AEs older than Vemurafenib’s FDA-approval (FAERS 2000-2011). The two graphs illustrate the corresponding drug (left) and target (right) side-effect profiles: circles’ radius reflect respective number of AEs in the dataset, blue-color represents AEs of Sorafenib (left) or of Sorafenib-specific targets (right), green-color represents AEs of Sunitinib (left) or of Sunitinib-specific targets (right), orange-color represents AEs of targets common to both drugs (right). Using the PRR characterization (x-axis) we can compare safety-profiles and identify known or novel relationships: e.g., while increased signals for Neoplasm- and Renal-disorders can be explained by the fact that both drugs are used for treating Renal Cell Carcinoma, Sorafenib is more associated to Hepatic- and Skin-reactions, as compared to Sunitinib. This side-effect dissimilarity could be attributed to differences in their molecular landscape (i.e., perturbation of targets specific to each drug may affect different molecular systems). Our approach allows generating molecular-level profiles and validate whether such molecular to clinical-phenotype relationships exist: the respective AE-profiles of these drugs’ targets (right) indicate that Sorafenib-specific targets are more associated with skin-reactions allowing thus hypothesize for the molecular-mechanism of dermatological reactions. It is reasonable to expect then that patients treated with *BRAF*-specific therapies may also experience such side effects. Indeed, the label of the *BRAF*-specific inhibitor Vemurafenib includes dermatological reactions.

**Figure S3. Virtual perturbation experiments**. Molecular profiling of phenotypes allows performing systematically virtual perturbation experiments by comparing target profiles. (a) One important parameter of this side-by-side investigation is the study of clinical features associated with any two entities, as derived from the molecular profiling of individual patient prescriptions. This approach enables to quickly develop testable molecular hypotheses regarding drug use. (b) This example’s study profile-diagram represents a virtual trial we conducted by measuring the disposition of death as outcome in two treatment groups of a skin cancer cohort – the results show that inhibition of beta-adrenergic receptors may improve mortality of skin cancer patients.
